# Obstetrics

**Published:** 1859-07

**Authors:** 


### OBSTETRICS.

1. ON COMPRESSION OF THE AORTA IN UTERINE HEMORRHAGE. By *Dr. Spiegelberg.*—M. Seutin, of Brussels, in a communication made to the Berlin Obstetrical Society, proposed what he termed a new method of arresting uterine hemorrhage, viz., the compression of the aorta, a procedure which he described as both easy of execution and certain of success. Of course, in a society of obstetricians, the pretension to novelty was soon disposed of, and the communication only calls for notice as having induced Dr. Spiegelberg to make some remarks condemnatory of the practice. That compression of the aorta will arrest uterine bleeding, he has convinced himself by many experiments; but the explanation of this is not due to the fact that all blood is thus prevented entering the uterus. The instant we divide the arteries conveying blood to the uterus, its muscular fibres contract, and the organ becomes diminished in size. Every one knows that such contraction will arrest hemorrhage. Dr. Spiegelberg has several times observed these remarkable results in both pregnant and non-pregnant animals; and has found the same consequence follow compression or ligature of the aorta, when it has passed the diaphragm. If, therefore, compression of the aorta thus excites powerful contraction of the uterus, it should evidently be an appropriate means for arresting hemorrhage, but the author's experiments show that for such compression to be of use, it must be permanent, the uterus distending again, as soon as the calibre of the artery becomes unobstructed. But on a living woman, compression could not be kept up continuously long enough to secure permanent contraction. Moreover, the aorta cannot be so completely compressed through the abdominal parietes, even immediately after delivery, as it can in animals when the cavity is laid open. So that, however surely we may seem to exert compression, some blood will always gain admission. Again, the closure of the aorta cannot be effected in woman as in animals, just below the diaphragm, but only after the large vessels of the intestines, and the renal and spermatic arteries have been given off; from which vessels the organ may obtain an abundant supply of blood. Lastly, the compression employed will also close the vena cava inferior; and the speaker's experiments and observations have convinced him that in such a case contraction of the uterus will not take place—the organ remaining then gorged with blood and relaxed. Indeed, it would *a priori* be expected that if the return of blood from the organ be obstructed, the vessels would become distended, and the open state of their orifices after birth would allow of the escape of their contents. This explains the cases in which R. Lee and Schneemann have observed the hemorrhage *increased* after compression of the aorta. Dr. Spiegelberg, therefore, regards the practice as destitute of a physiological basis, and of no practical utility; and he agrees with Dr. Schneemann, in considering the cases which have been seemingly benefited by it, to be really examples of excitement of contraction induced by the friction of the uterus made during the attempts at compression of the aorta.—(*Monatsschrift für Gebärtskunde*, Band xi. p. 26. *Medical Times and Gazette*, January 29, 1859.)

